# Tekt2 regulates hair cell development and function by maintaining normal kinocilium morphology in zebrafish

**DOI:** 10.3389/fcell.2026.1898887

**Published:** 2026-09-01

**Authors:** Biao Li, Po Xue, Jie Sun, Yubei Dai, Si Lu, Jie Gong, Dong Liu, Shuai Shi

**Affiliations:** 1 Department of Otolaryngology, Shanghai Pudong Hospital, Fudan University Pudong Medical Center, Shanghai, China; 2 School of Life Sciences, Nantong Laboratory of Development and Diseases, Co-innovation Center of Neuroregeneration, Nantong University, Nantong, China

**Keywords:** cell orientation, hair cell, kinocilium, mechanotransduction, TEKT2, zebrafish

## Abstract

**Background:**

Sensory hair cells (HCs) rely on a highly polarized apical apparatus, composed of actin-rich stereocilia and a microtubule-based kinocilium, to detect mechanical stimuli. Although tektins are conserved microtubule-associated proteins enriched in cilia and flagella, their roles in sensory HC morphogenesis and function remain poorly understood.

**Methods:**

We investigated the expression and function of tektin 2 (tekt2) in zebrafish HCs using single-cell RNA sequencing, whole-mount in situ hybridization, genetic loss-of-function and rescue analyses, behavioral assays, scanning electron microscopy, and FM-dye uptake assays.

**Results:**

Single-cell RNA sequencing and whole-mount in situ hybridization showed that tekt2 is highly expressed in neuromast, macular, and crista HCs. A tekt2-mCherry reporter further revealed enrichment of Tekt2 at the HC apex and within the kinocilium. The tekt2 deficiency caused auditory and vestibular dysfunction and disrupted otolith formation, accompanied by reduced and shortened otic cilia. In neuromasts and cristae, tekt2 deficiency decreased HC number, shortened hair bundles, and produced abnormal kinocilia. These kinocilium defects were associated with disorganized stereocilia and disrupted HC orientation. Mechanotransduction was consistently compromised, as indicated by reduced FM-dye uptake, and could be partially restored by exogenous tekt2.

**Conclusion:**

Together, our findings identify Tekt2 as an HC-enriched microtubule-associated factor required for normal kinocilium morphology, apical polarity, hair-bundle organization, and mechanotransduction function in zebrafish HCs.

## Introduction

Hearing loss and vestibular dysfunction are the two most common inner-ear disorders that profoundly impair human quality of life. The inner ear is the primary sensory organ for hearing and balance, comprising the cochlea and the vestibular system (Hudspeth, 2014). HCs are the primary sensory receptors in both systems, translating mechanical stimuli into neural activity (Qi et al., 2024). Cochlear HCs respond to sound-induced mechanical vibrations by converting them into electrical signals, which are transmitted to the brain. In contrast, vestibular HCs detect linear and angular accelerations to maintain posture and gaze stability (Caprara and Peng, 2022). These functions depend on a specialized apical structure that each HC bears a bundle of actin-rich stereocilia arranged in a staircase pattern. Mechanical force is transmitted through tip links that connect neighboring stereocilia. When the bundle deflects toward the tallest row, tension in the tip links increases, opening mechanosensitive ion channels near the stereocilia tips and allowing K^+^ and Ca^2+^ influx (Howard et al., 1988; Moreland and Bird, 2022). This mechanoelectrical transduction process converts mechanical inputs into electrical signals that are relayed through afferent nerves to the central nervous system, enabling normal hearing and vestibular function (Zheng and Holt, 2021). In addition to stereocilia, each HC also has a microtubule-based kinocilium. In mammals, vestibular HCs retain the kinocilia after birth, whereas cochlear HCs lose them once planar cell polarity (PCP) is established (Leibovici et al., 2005). The kinocilium is a single, asymmetrically positioned cilium next to the stereocilia bundle, supported by a canonical axonemal microtubule scaffold (Gerdes et al., 2009; Liu et al., 2023a). It acts as a spatial reference that orients bundle polarity and biases the HC sensitivity to deflection in a particular direction. Accordingly, abnormal kinocilium positioning or misoriented migration is often linked to disrupted HC polarity and sensory impairment, highlighting the kinocilium as an important structural determinant of HC function (Wang and Zhou, 2021).

Tektins (Tekts) are highly conserved microtubule-binding proteins that are enriched in microtubule-based structures such as flagella and motile cilia, where they help preserve structural stability and normal function (Amos, 2008). In mammals, at least five Tekt proteins (Tekt1-Tekt5) have been described. Individual Tekt filaments are thought to form by the head-to-tail assembly of about 16 nm subunits (Pirner and Linck, 1994). Each Tekt subunit contains a central rod-like region built from four α-helical segments (1A, 1B, 2A, and 2B) connected by three linker regions (L1, L12, and L2). These helices fold back in a repeated pattern, creating an elongated, flexible scaffold (Gui et al., 2021). In mammals, Tekt1-Tekt5 are required for normal sperm flagellar structure and motility (Linck et al., 1982). Tekt1 is enriched at the centrosome and developing tail region of spermatids, consistent with a role in axoneme initiation and basal body assembly (Linck et al., 1982; Li et al., 2022). Tekt2 localizes along the axoneme, whose loss disrupts inner dynein arm–associated structures, leading to bent flagella, reduced motility, and male infertility in mice (Yamaguchi et al., 2014). Although the Tekt3-deficient male mice remain fertile and produce morphologically normal sperm, their sperm exhibit reduced motility with axonemal bending defects (Roy et al., 2009; Liu et al., 2023b). Tekt4 deficiency causes subfertility, accompanied by reduced sperm ATP levels and impaired motility coordination (Roy et al., 2007). Tekt5 localizes to the inner surface of the mitochondrial sheath in the sperm midpiece and has been implicated in supporting normal motility (Murayama et al., 2008).

Although these studies establish key roles for Tekts in flagella, their functions and mechanisms in other ciliary contexts remain less well defined. Tekts have also been linked to broader microtubule-dependent processes. Tekt4 and Tekt5 have been implicated in cancer cell behavior, including survival and metastatic potential. For example, Tekt4 knockdown suppresses proliferation and clonogenic growth in papillary thyroid carcinoma cells and is associated with reduced PI3K signaling activity (Zheng et al., 2018). Tekt5 has been reported to influence cell-cycle progression, in part by modulating tubulin stability and Hdac6 expression (Aoki and Matsui, 2019). Tekt2 has also been described as a microtubule-associated factor during mitosis, where it contributes to cytokinesis and proper cytoplasmic partitioning (Durcan et al., 2008). Together, these findings portray the Tekt family as microtubule-associated proteins that support the stability and performance of microtubule-based organelles and, in some settings, influence proliferation and signal transduction (Zheng et al., 2018; Xiahou et al., 2025).

Cilia are present on many cell types, including inner ear HCs, where they help cells sense and transduce external stimuli (Mill et al., 2023). Recent evidence indicates that *Cep78* regulates *Tekt* expression via a ubiquitin-dependent pathway, and that loss of *Cep78* disrupts sperm number and impairs hearing function, consistent with the clinical features of *CEP78* deficiency (Zhang et al., 2022). In zebrafish, disruption of *tekt3* has also been linked to hearing impairment, further implicating other *tekt* family genes in auditory function (Su et al., 2025). Based on our single-cell transcriptomic analyses, we found that *tekt2* is enriched in zebrafish HCs and is required for normal auditory and vestibular function. The *tekt2* mutants display abnormal otic vesicle morphology, increased HC loss, disorganized hair bundles, misoriented kinocilia, and impaired mechanosensory function. Together, these structural and polarity abnormalities are associated with compromised auditory and vestibular behaviors, highlighting an essential role for *tekt2* in HC morphogenesis and function.

## Results

### *Tekt2* is enriched in sensory HCs

**FIGURE 1:**
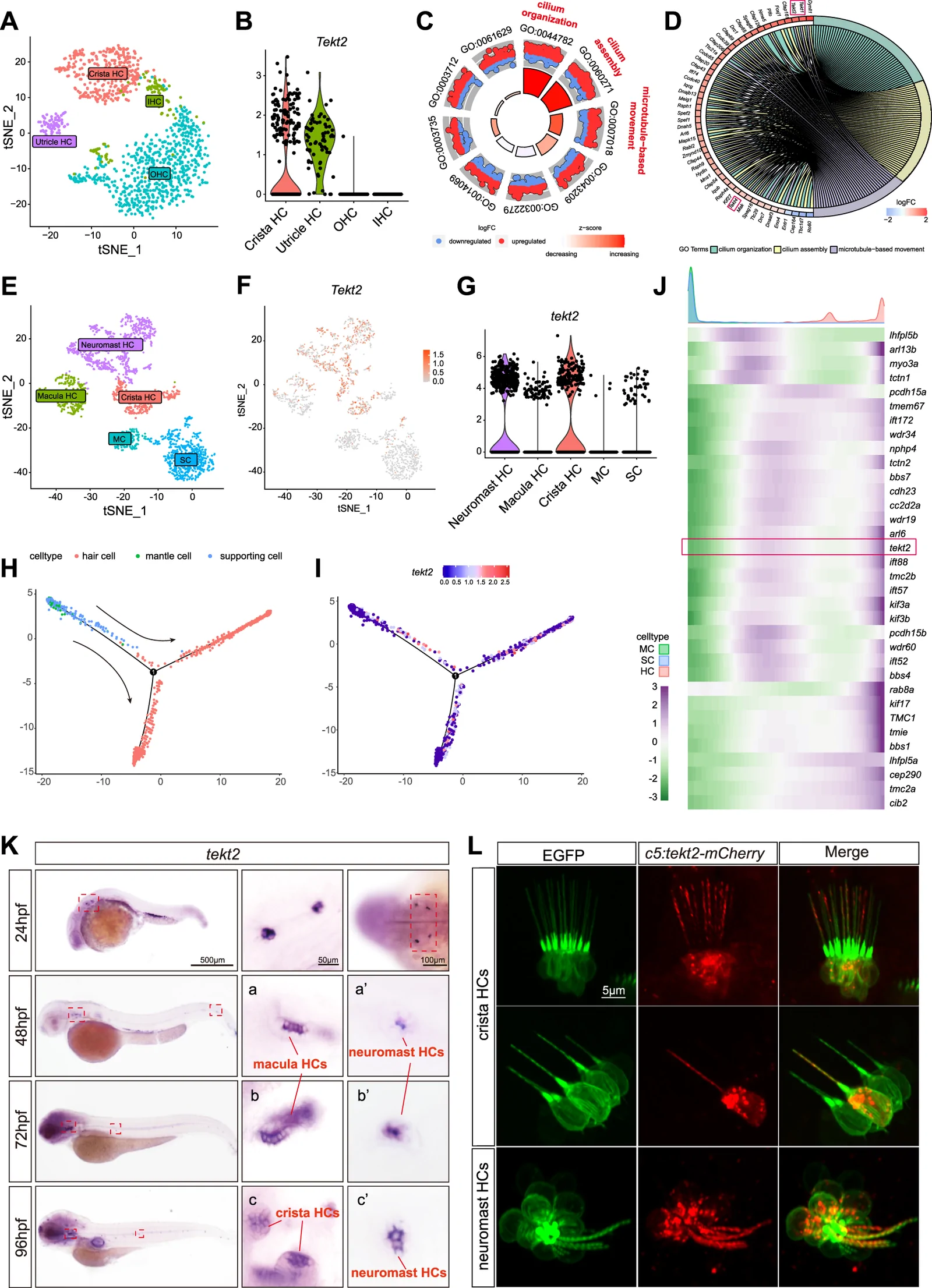
The expression profile of *tekt2*. **(A)** The t-SNE plot of different kinds of HCs in mice. **(B)** Violin plots showing the *tekt2* expression in different HC types of mice. **(C)** GO enrichment analysis of highly expressed genes in vestibular HCs. **(D)** Circos chord plot showing the top 50 genes enriched in the three most overrepresented GO terms identified in panel C (cilium organization, cilium assembly, and microtubule-based movement). **(E)** The t-SNE plot of different kinds of HCs and SCs in zebrafish. **(F,G)** Feature and violin plots showing the *tekt2* expression in different types. **(H,I)** Pseudotime analysis of *tekt2* expression profiles from SCs to HCs in zebrafish. **(J)** The heatmap showed the pseudo-time trajectories of the top marker genes during the SC to HC differentiation process. **(K)** The WISH analysis of *tekt2* expression in 24–96 hpf embryos. **(L)** Confocal imaging showed the expression of Tekt2 in HCs.

We integrated recently published single-cell transcriptomic datasets of mouse auditory and vestibular HCs (McInturff et al., 2018; Kolla et al., 2020; Wilkerson et al., 2021). Using four HC subtypes, we visualized cell states by t-SNE and assessed gene expression by feature and violin plots. *Tekt2* was robustly expressed in vestibular HCs but was nearly undetectable in cochlear HCs (Figures 1A,B). To define molecular programs that distinguish vestibular from cochlear HCs, we performed GO enrichment analysis on vestibular-high genes. These genes were strongly enriched for cilium- and microtubule-related processes, including cilium organization, cilium assembly, and microtubule-based movement (Figure 1C). Notably, *Tekt2* ranked among the top candidates contributing to these pathways (Figure 1D).

We next analyzed our zebrafish scRNA-seq dataset generated from GFP-positive cells purified by fluorescence-activated cell sorting (FACS) from *Tg(pou4f3:mEGFP)* larvae. *tekt2* was broadly expressed across zebrafish HCs, with particularly high expression in neuromast and crista HCs (Figures 1E–G). Pseudotime analysis further revealed a dynamic *tekt2* expression pattern, with elevated levels during the transition from supporting cells to HCs and again during later maturation, mirroring the trajectory of core ciliogenesis genes such as *ift88* (Figures 1H–J). To validate the developmental expression pattern, we performed WISH across multiple embryonic stages. *tekt2* transcripts were detectable in macular HCs at 24 hpf and remained consistently enriched in macular, crista, and neuromast HCs from 48 to 96 hpf (Figure 1K), in agreement with the single-cell data. Finally, we generated a HC reporter in which the c5h11orf1 promoter drives a Tekt2–mCherry fusion protein. Confocal imaging showed that the mCherry signal was enriched at the apical region of HCs and co-localized with the GFP-labeled kinocilium (Figure 1L).

### Loss of *tekt2* caused developmental abnormalities and body curvature in zebrafish

**FIGURE 2:**
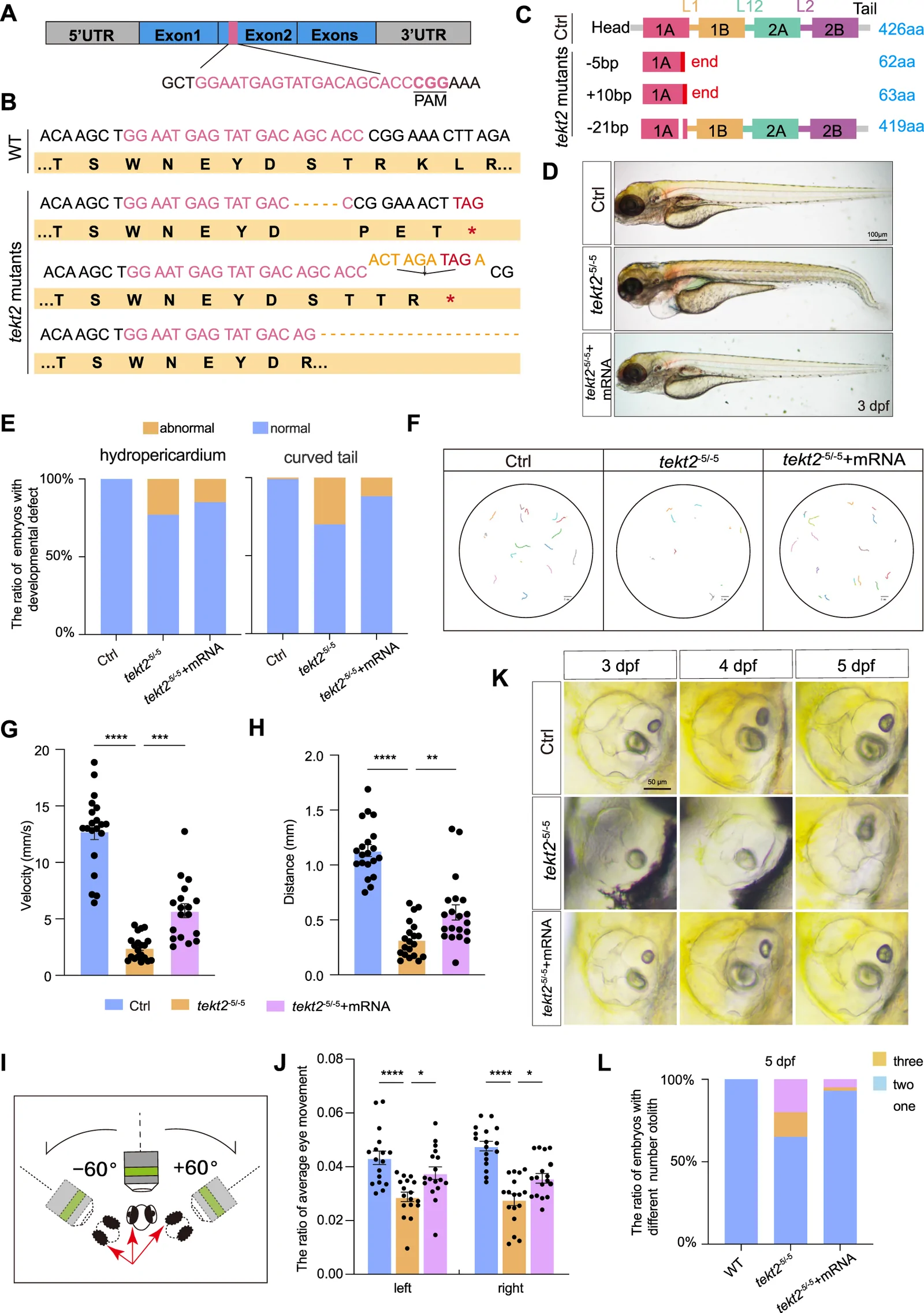
*tekt2* deficiency resulted in abnormal otolith formation and auditory dysfunction in zebrafish. **(A)** Schematic diagram of the *tekt2* gene structure and the corresponding gRNA target sites. **(B)** Sanger sequencing analysis of the *tekt2* F1 mutant reveals three distinct genotypic variants. **(C)** Schematic diagram illustrating the protein domains of the three distinct mutants. **(D)** Representative images of control and mutant zebrafish at 3 dpf. **(E)** Quantification of embryos with developmental defects (n = 100, *n* represents biologically independent zebrafish, with each fish considered as one experimental unit). **(F)** Swimming trajectories of 5 dpf zebrafish from the control, *tekt2* mutants, and rescue group. **(G,H)** Quantification of startle response distance and peak velocity (n = 20). **(I)**. Schematic illustration of the VOR assay in zebrafish. **(J)** Quantification of eye movement in the control, *tekt2* mutants, and rescue group (n = 17). **(K)** Imaging results of the otic vesicle and otoliths in control, *tekt2* mutants, and rescue groups at different developmental stages. Black dashed lines mark the boundaries of the otic vesicles, and red dashed lines mark the boundaries of the otoliths. Representative images of three independent experiments. **(L)** Quantification of embryos with different numbers of otoliths (n = 100). *p < 0.05, **p < 0.01, ***p < 0.001, ****p < 0.0001. All quantitative data were presented as mean ± SEM.

To examine the role of *tekt2* in zebrafish, we generated *tekt2* mutants in the *Tg(pou4f3:mEGFP)* background using CRISPR/Cas9, in which the HC membrane is labeled by EGFP. The sgRNA target site was designed within exon 2 (Figure 2A). Screening of F1 offspring identified three predominant indel alleles (−5 bp, +10 bp, and −21 bp) (Figure 2B). Both the −5 bp and +10 bp alleles are predicted to introduce premature stop codons. We selected the −5 bp allele and established a stable homozygous *tekt2* mutant line for subsequent analyses. Relative to the wild-type protein, the predicted mutant Tekt2 is truncated and lacks several conserved regions, including domains 1B, 2A, and 2B, which are expected to compromise Tekt2 structural integrity and interactions within the Tekt family (Figure 2C). We next assessed early developmental phenotypes and found that by 3 dpf, some *tekt2* mutants exhibited overt morphological abnormalities, including body curvature and pericardial edema (Figure 2D). To quantify penetrance, we randomly scored 100 embryos without morphological preselection and found that more than 20% of mutants displayed hydropericardium, and up to 30% showed curved body morphology (Figure 2E). Injection of exogenous *tekt2* mRNA partially rescued these defects, supporting a specific requirement for *tekt2* during early development. Importantly, homozygous mutant zebrafish with the normal developmental phenotypes were viable to adulthood and fertile. For the subsequent analyses of HC development and behavioral tests, we specifically used homozygous mutant larvae with grossly normal morphology.

### Loss of *tekt2* impaired auditory and vestibular behaviors and disrupted otolith development

Given the strong enrichment of *tekt2* in HCs, we asked whether the deficiency of *tekt2* compromised sensory function. We first assessed auditory function using an acoustic startle-response assay. Within the first 6 s after stimulation, *tekt2* mutants displayed markedly reduced locomotor activity compared with controls (Figure 2F). Quantification revealed significant decreases in total distance and peak velocity (Figures 2G,H). These deficits were partially rescued by injection of exogenous *tekt2* mRNA, supporting the specificity of the phenotype. We next evaluated vestibular function using the VOR assay. Following 60° rotational stimulation, eye movements were recorded as a readout of vestibular integrity (Figure 2I). *tekt2* mutants showed significantly reduced eye-movement amplitudes in both eyes, and this defect was alleviated by *tekt2* mRNA injection (Figure 2J). We also examined otic vesicle morphology and found that *tekt2* mutants revealed pronounced otolith defects, including ectopic positioning and abnormal otolith number, with frequent deviations from the normal two-otolith configuration to one or three otoliths (Figures 2K,L).

### Loss of *tekt2* disrupted cilia development in the otic vesicle

**FIGURE 3:**
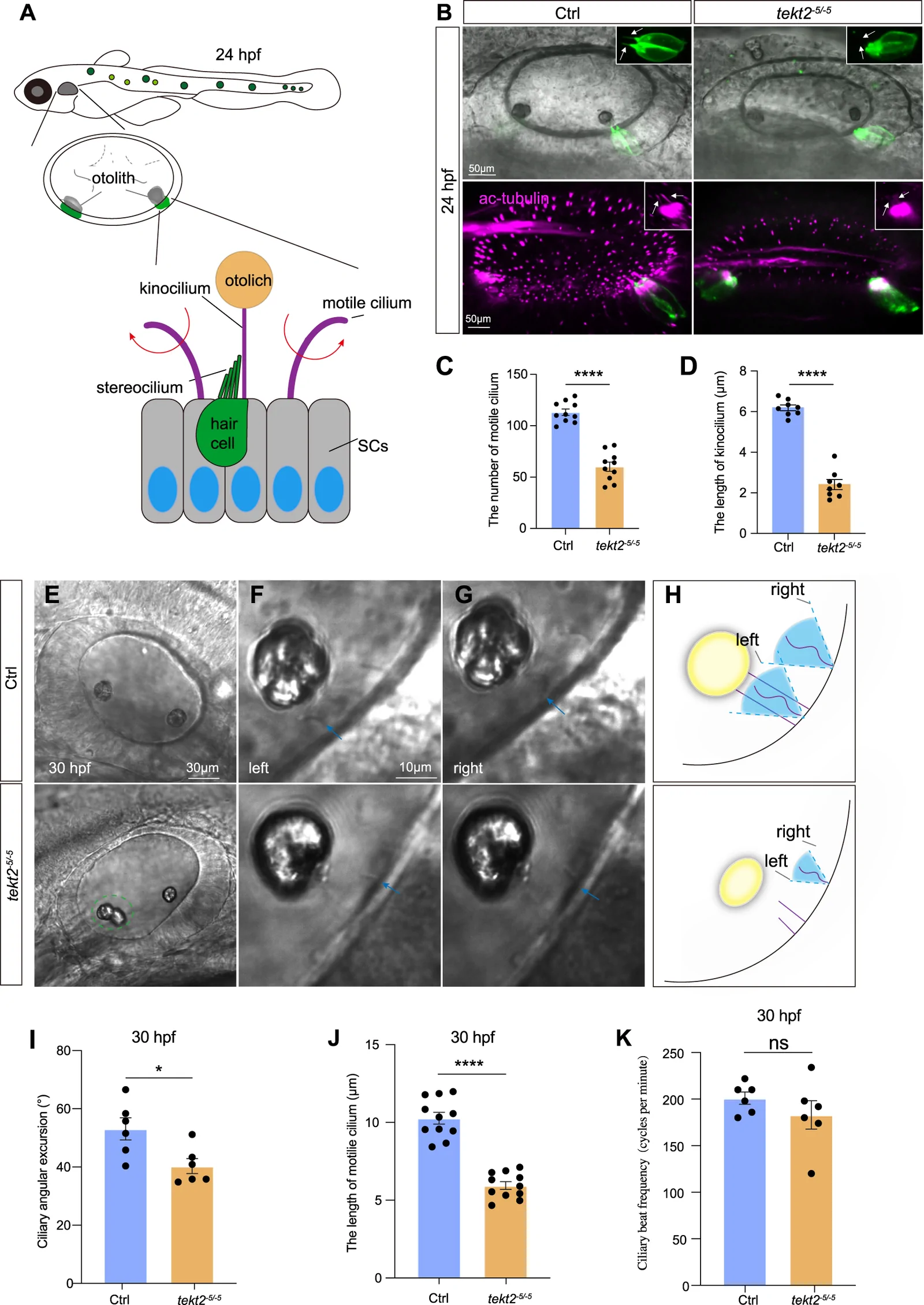
*tekt2* deficiency resulted in ciliary dysplasia in the zebrafish otic vesicle. **(A)** Schematic diagram of the cilia in the zebrafish otic vesicle. **(B)** Confocal imaging analysis of macula HCs and the motile cilia in the otic vesicle of 24 hpf zebrafish. **(C)** Quantification of the number of motile cilia (n = 10, *n* represents biologically independent zebrafish, with each fish considered as one experimental unit). **(D)** Quantification of the kinocilium length (n = 8). **(E-G)** Imaging results of the otic vesicle and otoliths in control and *tekt2* mutants at 30 hpf. Blue arrows indicate motile cilia. Representative images of three independent experiments. **(H)** Schematic diagram illustrating the beating pattern of motile cilia adjacent to the otolith primordium. The ciliary beating range is defined as the span between the leftmost and rightmost positions reached by the cilium. **(I–K)** Quantification of ciliary angular excursion(n = 6), motile cilia length (n = 11), and ciliary beat frequency (n = 6). *p < 0.05, ****p < 0.0001. All quantitative data were presented as mean ± SEM.

In the zebrafish inner ear, normal otolith formation and positioning require both macular HC kinocilia, which tether precursor particles at the seeding site, and motile cilia on the otic vesicle surface that generate local fluid flow to transport these particles (Figure 3A). To define the cellular basis of the otolith defects in *tekt2* mutants, we immunolabeled otic cilia with an anti-tubulin antibody and performed confocal imaging. At 24 hpf, *tekt2* mutants showed a pronounced reduction in the number of motile cilia within the otic vesicle (Figures 3B,C). Importantly, although macular HCs were present, their tethering kinocilia were markedly shortened or absent in mutants (white arrows; Figures 3B,D), providing a plausible structural explanation for otolith mislocalization. At 30 hpf, *tekt2* mutants retained unfused otoliths compared with the control zebrafish (green circles; Figure 3E). We next recorded otic vesicle dynamics and found that, compared with the control zebrafish, *tekt2* mutants displayed compromised ciliary movement (Supplementary Movie 1). To quantify motile cilia dynamics, we measured ciliary motion within the region surrounding the otoliths. Ciliary beat amplitude was defined as the displacement range between the leftmost and rightmost positions relative to the midline, and representative frames at these extremes are shown (blue arrows; Figures 3F–H). The results showed that *tekt2* mutants exhibited a reduced ciliary angular excursion and shorter cilia compared with controls (Figures 3I,J). In addition, no significant difference in the apparent ciliary beat frequency was detected between control and *tekt2* mutants under the imaging conditions used (Figure 3K).

### Loss of *tekt2* disrupted HC development in cristae and neuromasts

**FIGURE 4:**
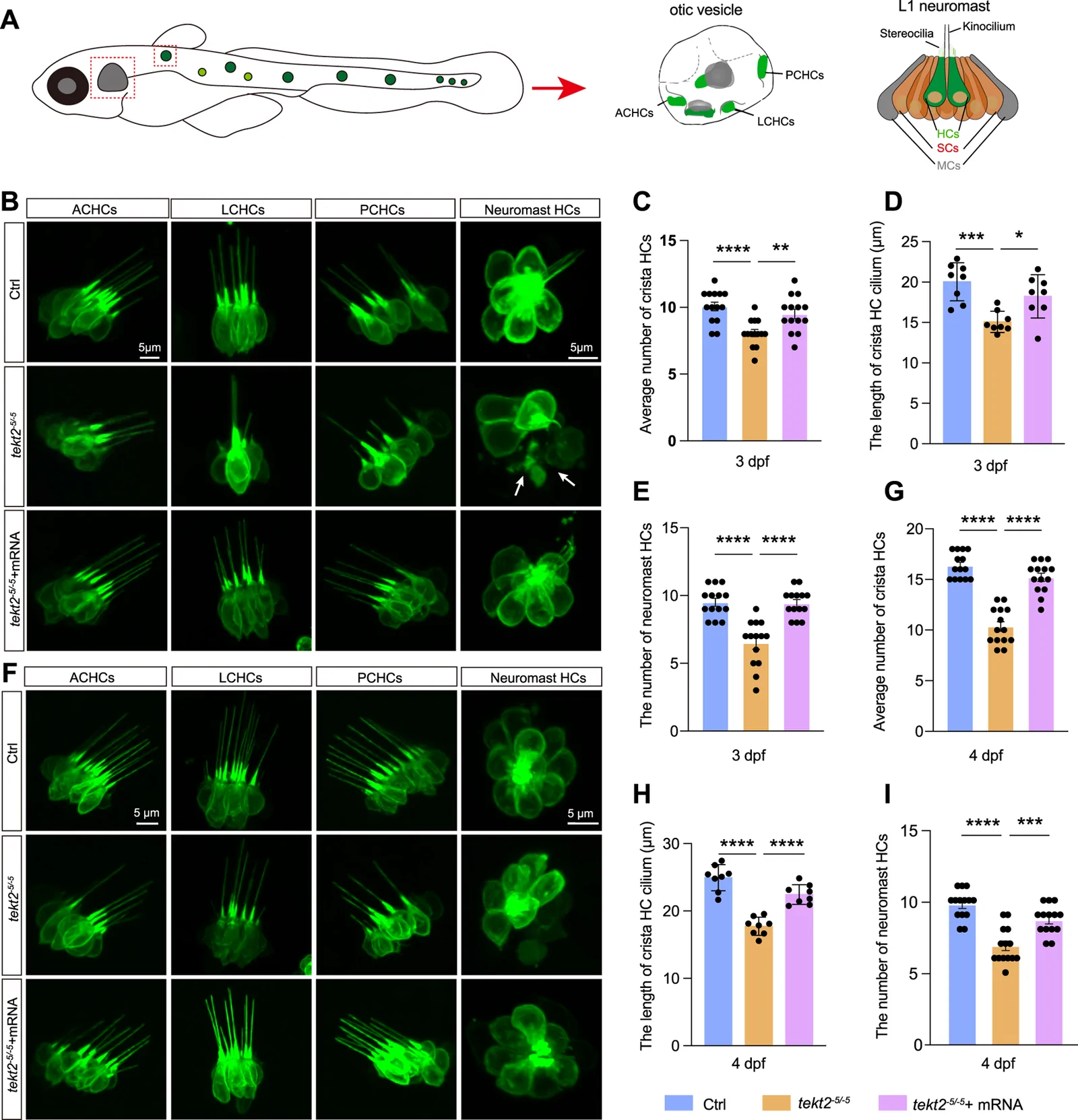
Loss of *tekt2* reduced the number of crista and neuromast HCs. **(A)** Schematic diagram of the location of crista and neuromast HCs in zebrafish. **(B,F)** Confocal images of crista and neuromast HCs in the control, *tekt2* mutants, and rescued zebrafish at 3 and 4 dpf. **(C–E)** Quantification of the number of crista HCs **(C)**, n = 14, *n* represents biologically independent zebrafish, with each fish considered as one experimental unit), the length of crista HC cilia **(D)**, n = 8), and the number of neuromast HCs **(E)**, n = 14) at 3 dpf. **(G–I)** Quantification of the number of crista HCs **(G)**, n = 14), the length of crista HC cilia **(H)**, n = 8), and the number of neuromast HCs **(I)**, n = 14) at 4 dpf. *p < 0.05, ***p < 0.001, ****p < 0.0001. All quantitative data were presented as mean ± SEM.

Because *tekt2* is highly enriched in crista and neuromast HCs, we quantified HC numbers in the crista and L1 lateral-line neuromast region (Figure 4A). The results showed that *tekt2* mutants exhibited a significant decrease in neuromast HCs compared to the control zebrafish at 3dpf (Figures 4B,E). We next examined crista HCs and observed a comparable deficit. At 3 dpf, *tekt2* mutants displayed significantly fewer HCs in all three cristae (Figures 4B,C). In addition, the kinocilia of crista HCs were markedly shortened in mutants (Figure 4D). These neuromast and crista phenotypes persisted at 4 dpf and were partially rescued by injection of exogenous *tekt2* mRNA (Figures 4F–I), supporting a specific requirement for *tekt2* in HC development and kinocilium integrity.

### *tekt2* knockdown impairs otolith development, HC formation, and sensory behaviors

**FIGURE 5:**
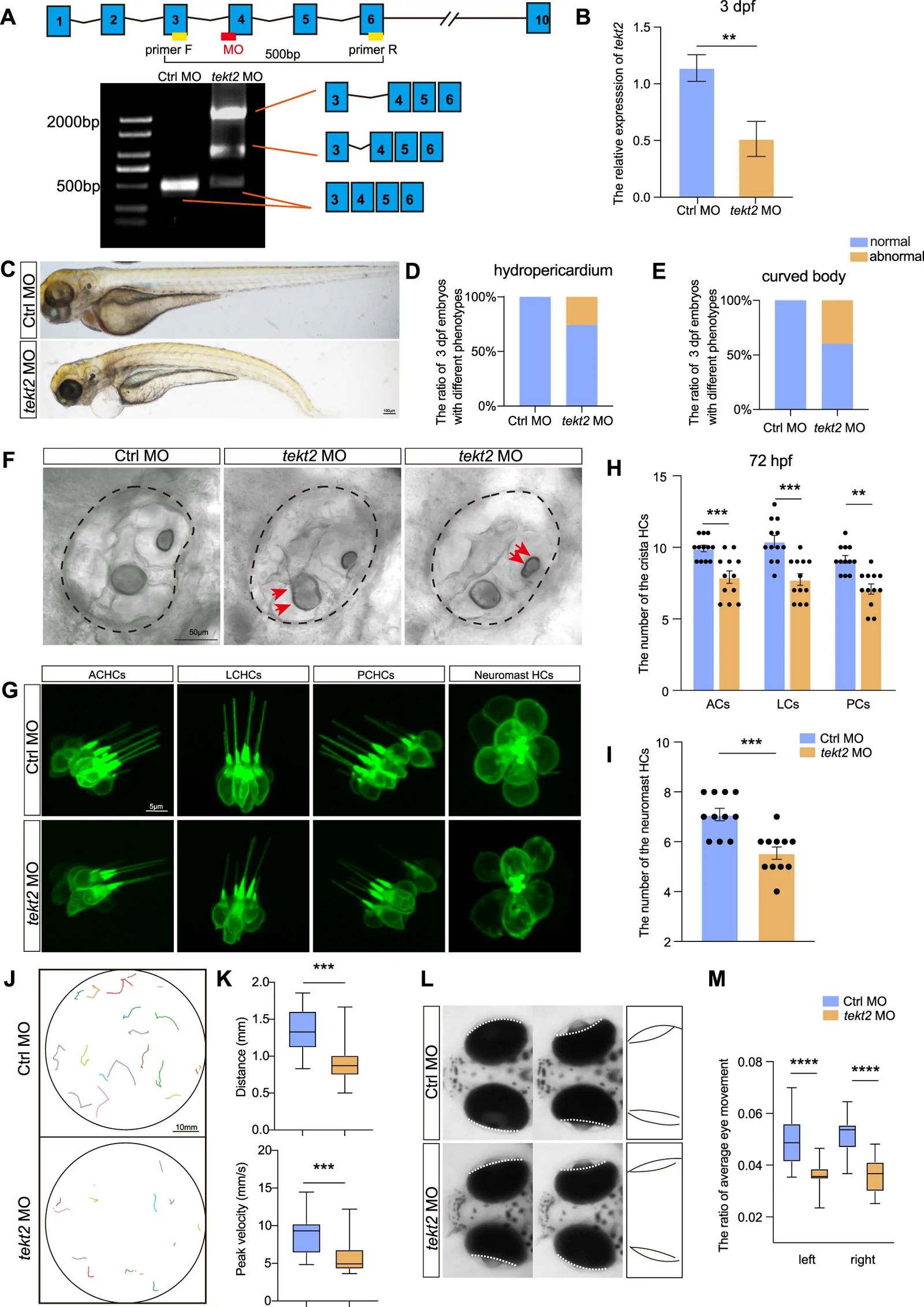
Knockdown of *tekt2* causes defects in otic vesicle development, HC formation, and sensory-associated behaviors. **(A)** The diagram of the *tekt2* MO acting site and the Gel electrophoresis results revealed that aberrant splicing of the *tekt2* sequence led to the transcription of two erroneous sequences, significantly reducing the expression of the correctly spliced transcript. **(B)** The qRT-PCR showing reduced *tekt2* mRNA expression in *tekt2* morphants. **(C)** Representative images of control and *tekt2* morphant. **(D)** Quantification of embryos with hydropericardium (n = 100, *n* represents biologically independent zebrafish, with each fish considered as one experimental unit). **(E)** Quantification of embryos with curved body (n = 100). **(F)** Imaging results of the otic vesicle and otoliths in control and *tekt2* morphants. Black dashed lines marked the boundaries of the otic vesicles, and red arrows marked the abnormal otoliths. Representative images of three independent experiments. **(G)** Confocal images of crista and neuromast HCs in the control zebrafish and *tekt2* morphants at 3 dpf. **(H,I)** Quantification of the number of crista HCs (H, n = 12) and neuromast HCs (I, n = 11) at 3 dpf. **(J)** Swimming trajectories of 5 dpf zebrafish from the control and *tekt2* morphants. **(K)** Quantification of startle response distance and peak velocity (n = 20). **(L)** The imaging showing the eye rotation in control and *tekt2* morphants of the VOR assay. **(M)** Quantification of eye movement in control and *tekt2* morphants (n = 17). **p < 0.01, ***p < 0.001, ****p < 0.0001. All quantitative data were presented as mean ± SEM.

To confirm that the phenotypes observed in *tekt2* mutants are attributable to loss of *tekt2*, we reduced *tekt2* expression using a splice-blocking morpholino (MO) injected into one-cell-stage *Tg(pou4f3:mEGFP)* embryos. RT-PCR confirmed aberrant splicing of *tekt2* pre-mRNA in MO-injected embryos, producing two mis-spliced products caused by intron retention, while the correctly spliced transcript was markedly reduced (Figure 5A). qPCR further demonstrated a significant decrease in *tekt2* mRNA levels after MO injection (Figure 5B), confirming effective knockdown. Phenotypic analysis showed that *tekt2* morphants recapitulated key developmental abnormalities observed in mutants. Approximately 26% of morphants developed pericardial edema, and up to 40% displayed abnormal trunk curvature (Figures 5C–E). Confocal imaging further revealed impaired fusion of otoliths (red arrows; Figure 5F) and a marked reduction in HC numbers in both cristae and neuromasts (Figures 5G,I). Consistent with these structural defects, *tekt2* knockdown also caused measurable sensory impairment. In the acoustic startle-response assay, *tekt2* morphants exhibited reduced locomotor trajectories following stimulation, with significantly decreased total distance and peak velocity compared with controls (Figures 5J,K), indicating compromised auditory performance. Moreover, VOR measurements showed reduced eye-movement amplitudes in *tekt2* morphants compared to controls (Figures 5L,M).

### Loss of *tekt2* disrupted kinocilium morphology and HC orientation in neuromast

**FIGURE 6:**
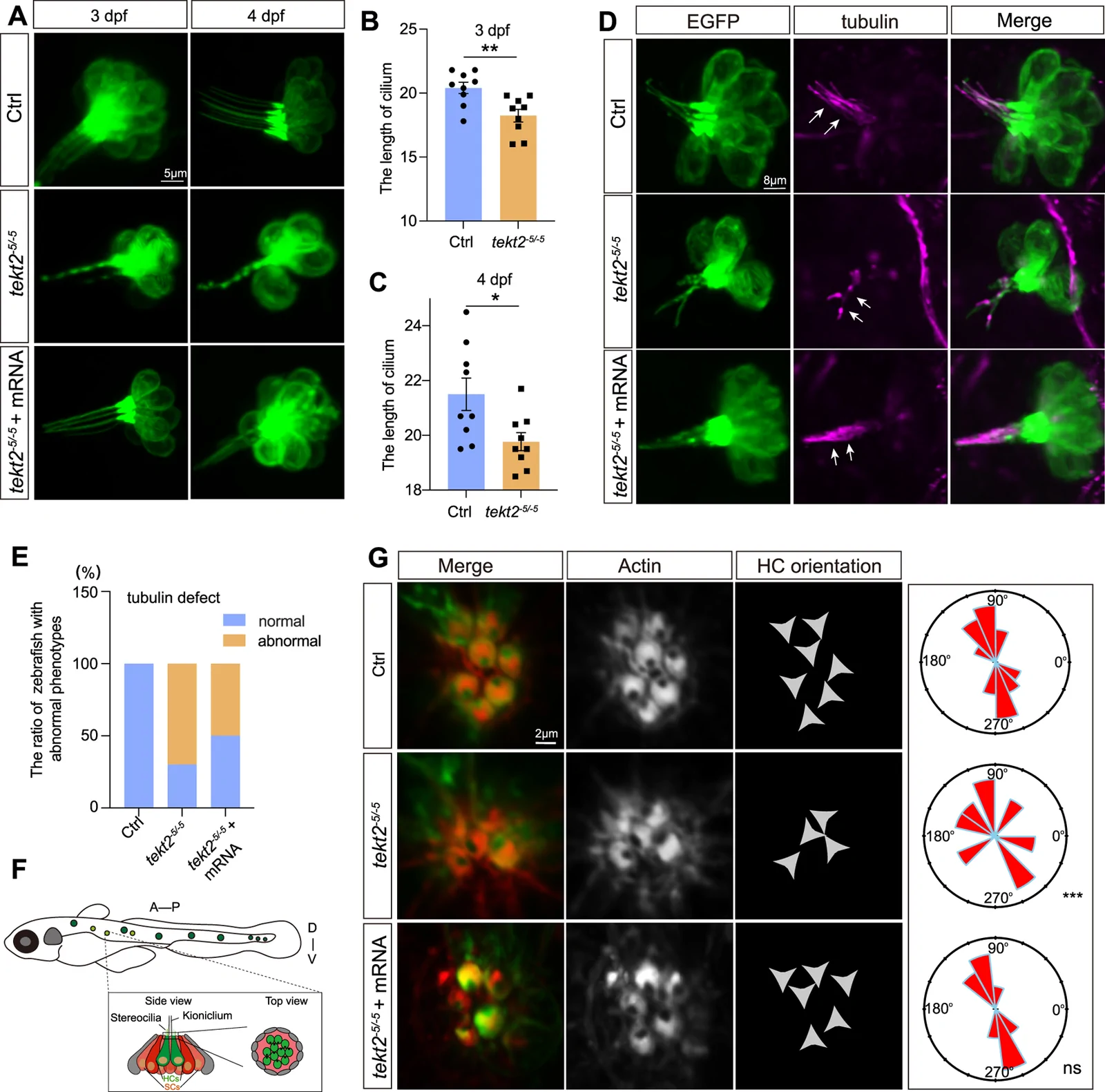
Loss of *tekt2* disrupted kinocilium morphology and HC orientation. **(A)** Confocal images of neuromast HCs (lateral view) in the control, *tekt2* mutants, and rescued zebrafish at 3 and 4 dpf. **(B,C)** Quantification of the length of neuromast HC kinocilium at 3 and 4 dpf (n = 9, *n* represents biologically independent zebrafish, with each fish considered as one experimental unit). **(D)** Confocal images of neuromast HCs (lateral view) stained with tubulin in the control, *tekt2* mutants, and rescued zebrafish at 4 dpf. Arrows point to the kinocilia. **(E)** Quantification of embryos with kinocilia defects (n = 100). **(F)** Diagram of the zebrafish showing the two different orientations of neuromast HCs. **(G)** Phalloidin staining of secondary neuromast HCs of control, *tekt2* mutants, and rescued zebrafish at 4 dpf. Representative images of three independent experiments. *p < 0.05, ***p < 0.001. All quantitative data were presented as mean ± SEM.

We then examined the integrity of kinocilia in lateral-line neuromast HCs. At 3 and 4 dpf, neuromast HCs in controls displayed well-organized bundles with continuous GFP-labeled kinocilia, whereas *tekt2* mutants exhibited fragmented kinocilia with discontinuous GFP signal and uneven fluorescence distribution (Figure 6A). We further immunolabeled kinocilia with an anti-tubulin antibody and observed aberrant tubulin accumulation in 70% of mutant embryos (Figures 6D,E). Importantly, these structural defects were substantially rescued by injection of exogenous *tekt2* mRNA (Figures 6A–E).

In the zebrafish lateral-line system, HCs in primary neuromasts are planar polarized along the anterior-posterior axis, whereas those in secondary neuromasts are aligned along the dorsal-ventral axis (Navajas Acedo et al., 2019). Within each neuromast, paired HCs are oppositely oriented, with their kinocilia pointing toward each other along a common axis (Figure 6F). We next examined whether neuromast HC orientation was altered in tekt2 mutants by staining neuromasts with phalloidin to label F-actin in stereocilia. At 3 dpf, stereocilia appeared as actin-rich bundles, and the kinocilium position corresponded to the characteristic actin-free gap within each bundle. In controls, kinocilia were consistently positioned in opposite directions across paired HCs, whereas *tekt2* mutants displayed disorganized kinocilium positioning and loss of normal opposing orientation of paired HCs (Figure 6G). Exogenous *tekt2* mRNA restored kinocilium orientation and partially rescued the HC orientation arrangement defects. Together, these data indicate that *tekt2* is required for normal kinocilium development and for coordinated HC orientation in neuromast.

### Loss of *tekt2* impaired stereocilia development and mechanotransduction in lateral-line HCs

**FIGURE 7:**
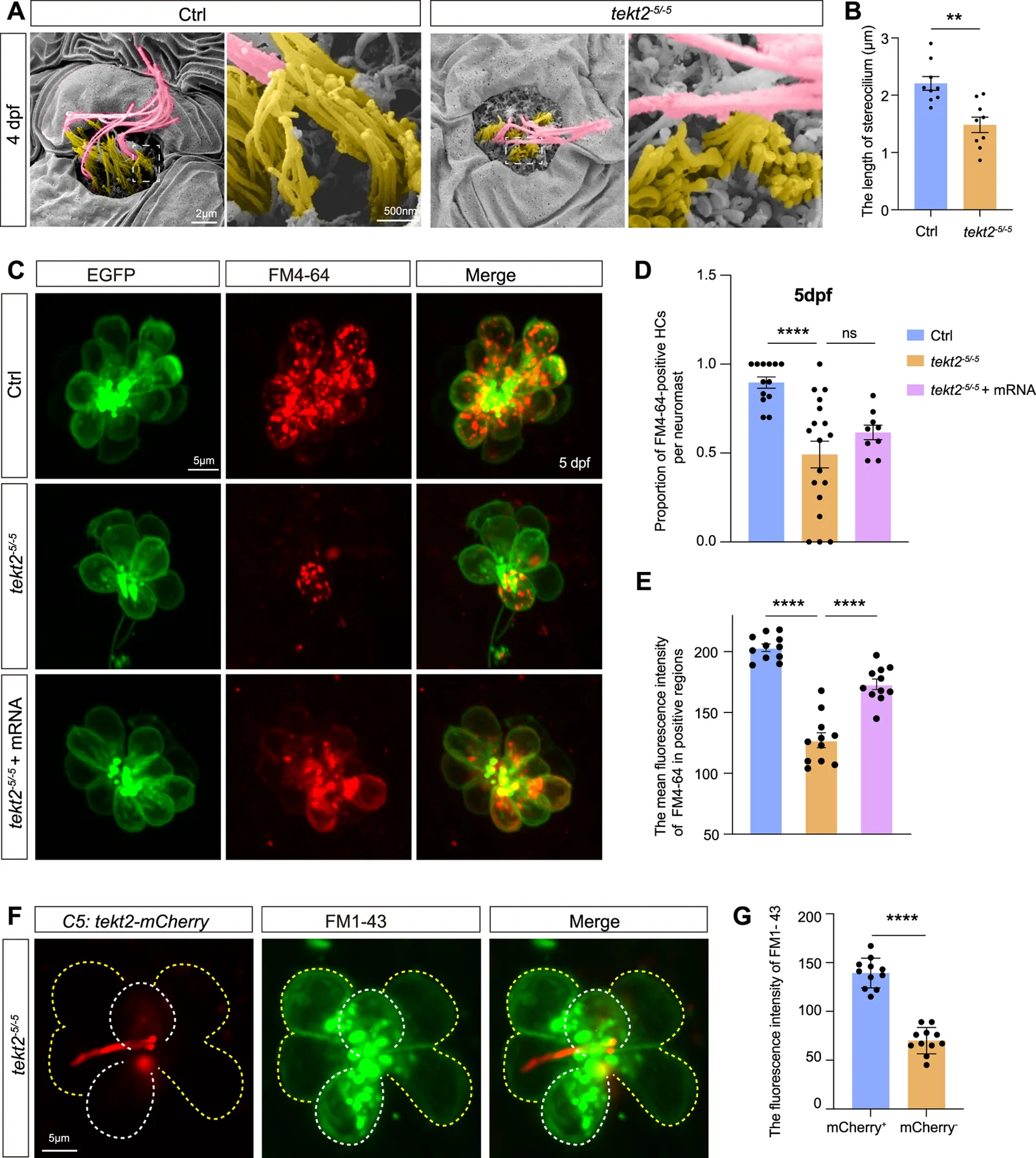
The *tekt2* mutation resulted in the abnormal structure of stereocilia bundles and impaired MET function in zebrafish HCs. **(A)** SEM images of the HC stereocilia bundles in the control and *tekt2* mutants at 4 dpf. **(B)** Quantification of the length of neuromast HC stereocilia at 4 dpf (n = 9, *n* represents biologically independent zebrafish, with each fish considered as one experimental unit). **(C)** Confocal images of the 5 dpf zebrafish HCs treated with FM4-64 dye. **(D)** Quantification of the FM4-64 index of HC per neuromast (For ctrl, n = 13; for *tekt2* ^*−5/−5*^, n = 18; for *tekt2* ^*−5/−5*^+mRNA, n = 9). **(E)** Quantification of the mean FM4-64 fluorescence intensity within the dye-positive regions of each neuromast (n = 11). The integrated FM4-64 fluorescence intensity was divided by the corresponding dye-positive area. **(F)** Confocal imaging showing uptake of FM1-43 dye by HCs in the same neuromast of 5 dpf *tekt2* mutants after the injection of *tekt2-mCherry* fusion protein plasmid. **(G)** The statistical results of FM1-43 fluorescence intensity (n = 11). *p < 0.05, **p < 0.01, ****p < 0.0001. All quantitative data are presented as mean ± SEM.

Because kinocilium integrity contributes to proper stereocilia bundle assembly, we asked whether *tekt2* loss further disrupted stereocilia development. Scanning electron microscopy of neuromast HCs at 4 dpf showed that control animals displayed the expected staircase-like bundles, whereas a subset of HCs in *tekt2* mutants exhibited partial loss of graded bundle organization (Figure 7A). Quantification further revealed that the tallest stereocilia were significantly shorter in mutants than in controls (Figure 7B), indicating compromised hair-bundle growth and structural integrity. Because mechanotransduction (MET) channels are located on stereocilia, we next examined whether the observed hair-bundle abnormalities in *tekt2* mutants were accompanied by altered MET function. Styryl dyes such as FM4-64 rapidly enter HCs through open MET channels and are widely used as functional reporters of MET activity (Erickson et al., 2017). Therefore, we quantified MET competence as the fraction of FM4-64-positive cells among GFP-labeled HCs after this dye treatment. Confocal imaging showed that *tekt2* mutants had a significantly reduced proportion of FM4-64-positive HCs (Figures 7C,D). In addition, the mean FM4-64 fluorescence intensity within the dye-positive regions was significantly reduced in *tekt2* mutants (Figure 7E). Importantly, injection of exogenous *tekt2* mRNA partially restored FM4-64 uptake (Figures 7C–E), supporting a requirement for *tekt2* in maintaining normal MET channel activity. Furthermore, FM1-43 dye uptake by the HCs in *tekt2* mutants was detected after injection of the *tekt2-mCherry* fusion plasmid, and we found that HCs labeled with mCherry exhibited enhanced FM1-43 uptake ability in the mosaic zebrafish compared to adjacent HCs lacking mCherry (Figures 7F,G). These findings demonstrate that *tekt2* deficiency is associated with impaired MET function, as indicated by reduced rapid FM-dye uptake.

## Discussion

Tekts are a conserved family of microtubule-binding proteins that form part of the internal scaffold of axonemal microtubules in cilia and flagella. They are enriched in the axoneme and help preserve both structural integrity and motility, including proper organization of dynein-based motor assemblies that generate rhythmic bending and propulsion (Amos, 2008). Tekt2, in particular, localizes along the axoneme in sperm, and its loss disrupts dynein-associated architecture, leading to abnormal flagellar curvature, reduced motility, and male infertility (Yamaguchi et al., 2014). In the present study, integrative analyses of single-cell transcriptomic datasets from mouse auditory HCs revealed that *Tekt2* was highly enriched in vestibular HCs, particularly crista HCs, but is scarcely expressed in cochlear HCs. Moreover, GO enrichment of vestibular-high genes was dominated by cilium and microtubule-related terms, including cilium organization, cilium assembly, and microtubule-based movement, with *Tekt2* among the most strongly enriched genes. This aligns with a key anatomical difference between mammalian HC types: vestibular HCs retain the kinocilium after birth, whereas cochlear HCs lose them once planar polarity is established (Leibovici et al., 2005; Wang and Zhou, 2021). In zebrafish, *tekt2* transcripts were detected in nearly all HC populations, with especially strong expression in crista and neuromast HCs. Unlike mammals, all zebrafish HCs retain their kinocilia throughout life. Therefore, the expression profile of *tekt2* in zebrafish HCs further suggests its conserved role in kinocilium formation and maintenance across species. Pseudotime analysis further showed that *tekt2* follows an expression trajectory similar to that of core ciliogenesis genes, such as ift88, with high expression during the transition from SCs to HCs and during later maturation. Transgenic reporter assays confirmed strong localization of Tekt2 to the kinocilium. Because ift88 is critical for kinocilium formation and function (Kindt et al., 2012), the colocalization of Tekt2 with the kinocilium and its shared temporal expression pattern with ciliogenesis genes further support a link between Tekt2 and the ciliogenesis program.

Both the HC kinocilium and the motile cilia on the surface of the otic vesicle are rich in microtubules, and normal otolith formation depends on coordinated input from both ciliary populations (Stooke-Vaughan et al., 2012). Early in development, the first two macular HC kinocilia (tether cilia) tether the otolith nucleation. As development proceeds, otolith precursor particles, a type of calcium carbonate material, continue to accumulate on the otolith nucleus, forming otoliths with the correct position and shape (Stooke-Vaughan et al., 2012). In parallel, motile cilia on the otic vesicle surface beat in a directional pattern to generate fluid flow that helps deliver precursor particles to the otolith nucleation on both sides of the vesicle (Wu et al., 2011; Whitfield, 2020). In *tekt2* mutants, tether cilia were markedly shortened or absent by 24 hpf. This loss would be expected to weaken otolith nucleation anchoring and increase the likelihood of otolith misplacement. In addition, *tekt2* mutants also showed fewer and shorter motile cilia in the otic vesicle, along with a strong reduction in beating amplitude, which would likely impair the transport of otolith precursor particles and reduce effective fusion of these precursors. Together, these defects provide a plausible explanation for the abnormal otolith number and morphology observed in *tekt2* mutants. Moreover, we quantified the apparent ciliary beat rate and found no significant difference between control and tekt2 mutant embryos, with both groups showing values of approximately 200 cycles per minute (Figure 3K). However, these values were substantially lower than those previously reported for motile cilia in the zebrafish otic vesicle (Stooke-Vaughan et al., 2012). This discrepancy likely reflects the limited temporal resolution of the present recordings, as the acquisition rate of 15.2 frames per second was insufficient to accurately resolve the rapid beating frequency reported for zebrafish otic motile cilia. Therefore, the measured values may be affected by temporal aliasing and should be regarded as apparent beat rates rather than accurate measurements of the true ciliary beat frequency.

In this study, *tekt2* mutants exhibited weakened startle responses and reduced VOR performance, indicating impaired auditory and vestibular function in zebrafish. As the sensory receptors of the inner ear and the lateral line, HCs convert mechanical stimuli into electrical signals that the nervous system interprets to drive hearing, vestibular reflexes, and lateral-line sensation (Pichler and Lagnado, 2019). The deficiency of *tekt2* caused significant defects in both HC abundance and structure, which might further cause the auditory and vestibular dysfunction. Prior work suggests that tekts can influence basic cellular behaviors, including cellular migration, apoptosis, and proliferation. For example, suppression of Tekt5 has been reported to reduce germ-cell survival and increase apoptosis (Aoki and Matsui, 2019). In cancer models, TEKT4 has been linked to enhanced proliferation and metastatic potential of papillary thyroid carcinoma cells by activating PI3K/Akt signaling (Zheng et al., 2018). In zebrafish, the *tekt3* mutation delays neuromast HC regeneration through impairing the proliferation of non–HC populations (Su et al., 2025). In addition, *Tekt2* has also been reported to localize to spindle poles during metaphase and then relocalize to the central spindle during anaphase, where it is required for successful cytokinesis (Durcan et al., 2008). However, whether the reduced HC numbers in *tekt2* mutants arise predominantly from suppressed proliferation or increased apoptosis remains unresolved and warrants further investigation.

Beyond the reduction in HC number, the *tekt2* mutation caused striking defects in the kinocilium. Because the apical hair bundle is the functional core of the HC, these abnormalities are likely to have direct consequences for mechanosensory performance. In the vestibular system, each HC bundle contains a single microtubule-based kinocilium and multiple rows of actin-rich stereocilia (Peng et al., 2011; McGrath et al., 2021). The kinocilium is positioned next to the tallest stereocilia row and serves as an early reference point, whereas stereocilia form the staircase array that contains MET channels. Proper kinocilium development and positioning are critical for establishing the spatial organization and functional integrity of the stereocilia bundle (Kindt et al., 2012). Accordingly, defective kinocilium formation, shortening, or mispositioning is often accompanied by disordered stereocilia length relationships, loss of the normal staircase pattern, reduced bundle stability, and impaired MET function (Wang and Zhou, 2021; Kaushik et al., 2025). Consistent with defects in kinocilia, scanning electron microscopy and confocal imaging revealed marked disarray in stereocilia length and typical staircase architecture, with impaired MET channel function in *tekt2* mutants. These findings support a model in which *tekt2* may maintain stereocilia organization and mechanotransduction indirectly by preserving kinocilium integrity.

Lateral-line neuromast HCs form oppositely oriented pairs, and the kinocilium occupies the actin-free “gap” adjacent to the phalloidin-labeled stereocilia bundle in a stereotyped direction (Navajas Acedo et al., 2019). In *tekt2* mutants, this directional organization was lost, with kinocilium orientation becoming variable, and the secondary neuromasts no longer showed the typical opposing alignment along the dorsal–ventral axis. These findings suggest that *tekt2* helps maintain the kinocilium positioning at the apical surface for polarity establishment and maintenance. In zebrafish, PCP is established by oppositely oriented pairs of HCs, which is essential for appropriate deflection responses and normal sensory function (Landin Malt et al., 2019). Disruption of PCP is commonly associated with reduced mechanosensitivity and can lead to hearing impairment. For example, loss of *Vangl2* prevents the correct alignment of stereocilia bundles along the cochlear axis, disrupts bundle geometry, compromises mechanotransduction, and results in severe hearing loss (Montcouquiol et al., 2006; Derrick et al., 2024). Therefore, the auditory and vestibular behavioral deficits observed in *tekt2* mutant zebrafish may be related, at least in part, to the accompanying abnormalities in kinocilium morphology, hair-bundle orientation, PCP, and mechanotransduction.

In summary, this study combines cross-species single-cell expression profiling, *in vivo* localization, and genetic manipulation to identify *tekt2* as a key regulator of HC development in zebrafish. Loss of *tekt2* impaired otolith morphogenesis and led to measurable sensory deficits, including hearing loss and vestibular dysfunction. Moreover, *tekt2* deficiency caused HC kinocilium structural and orientation defects that were accompanied by abnormal hair-bundle architecture and reduced MET function. These findings support the idea that tekt2 maintains bundle orientation and MET function competence to regulate hearing and vestibular function and highlight *tekt2* as a candidate factor connecting cilia stability to HC function.

## Materials and methods

### Zebrafish husbandry and strains

Adult zebrafish (*Danio rerio*) were maintained under standard husbandry conditions at the Nantong University Zebrafish Facility. Embryos were collected by natural spawning and incubated at 28.5 °C in E3 medium (5 mM NaCl, 0.17 mM KCl, 0.33 mM CaCl_2_, 0.33 mM MgSO_4_). When required for imaging, embryos were transferred at 20 hpf to E3 medium supplemented with 0.2 mM phenylthiourea (PTU) to suppress pigmentation. All animal procedures were performed in accordance with protocols approved by the Animal Care and Use Committee of Nantong University. The zebrafish lines used in this study included wild-type fish and *Tg(pou4f3:mEGFP)*, in which membrane-targeted GFP labels HC membranes. They were obtained from the China Zebrafish Resource Center (CZRC).

### sgRNA/Cas9 mRNA synthesis and microinjection*s*

*tekt2* mutants were generated on the *Tg(pou4f3:mEGFP)* backgrounds using CRISPR-Cas9 technology. The sgRNA target sequence for *tekt2* was 5′- GGA​ATG​AGT​ATG​ACA​GCA​CC(CGG)-3′ (PAM in parentheses). Cas9 mRNA was synthesized *by in vitro* transcription. Cas9 mRNA (300 ng/μL) and sgRNA (100 ng/μL) were co-injected into one-cell-stage embryos. Genotyping was performed by PCR and Sanger sequencing. The primers used for genotyping were: *tekt2* mut-F, 5′- GCT​CTG​CGT​AAC​GAA​ACG​AC-3′; *tekt2* mut-R, 5′-GCG​CAC​AGG​CTT​TCA​GAT​TC-3′.

### Morpholino and mRNAs microinjections

A splice-blocking morpholino antisense oligonucleotide (5′-CCT​CCT​TGG​ACT​TAA​CAA​TTT​TGT​G-3′) targeting *tekt2* was purchased from Gene Tools. An equal concentration of a standard non-targeting morpholino (5′-CCT​CTT​ACC​TCA​GTT​ACA​ATT​TAT​A-3′) was used as control in separate embryos of the same batch during each experiment. The *tekt2* morpholino was diluted to 0.3 mM and injected into one-cell-stage embryos. For *tekt2* mRNA synthesis, the *tekt2* open reading frame (ORF) was cloned into the pCS2+ vector. The resulting plasmid was linearized and used as a template for *in vitro* transcription with the mMESSAGE mMACHINE High Yield Capped RNA Transcription Kit (SP6) (Invitrogen). Purified capped *tekt2* mRNA (100–200 pg) was injected into one-cell-stage embryos.

### Whole-mount *in situ* hybridization

Whole-mount *in situ* hybridization (WISH) was performed as previously described (Dai et al., 2026). Briefly, DNA fragments of *tekt2* were amplified by PCR using the following primers: *tekt2* ISH-F: 5′-CGC​TCT​CAC​TCT​GTC​CAA​GG-3′ and *tekt2* ISH-R: 5′-ATC​ACA​AGA​GCA​CAG​GTG​CA-3′. PCR products were subcloned into the pGEM-T Easy Vector (A1360, Promega). Recombinant plasmids were linearized and used as templates for *in vitro* transcription with a DIG RNA Labeling Kit (11175025910, Roche) according to the manufacturer’s instructions. BM Purple (11442074001, Roche) was used as the alkaline phosphatase substrate to develop color. Images were acquired using a Nikon Ni-U upright microscope.

### RNA extraction, RT-PCR and quantitative real-time PCR

Larvae were anesthetized, homogenized in TRIzol reagent, and stored at −80 °C for 24 h before RNA isolation. Total RNA was extracted according to the manufacturer’s instructions (15596018, Invitrogen), and residual genomic DNA was removed by DNase I treatment. RNA concentration and purity were measured using a NanoDrop ND-2000 spectrophotometer (Thermo Fisher Scientific), and RNA integrity was evaluated by agarose gel electrophoresis. 2 μg total purified RNA was reverse-transcribed into cDNA using HiScript III RT SuperMix (R323-01, Vazyme).

RT-PCR was performed in a total reaction volume of 20 μL containing 10 μL 2 × Phanta Flash Master Mix (P520-01, Vazyme) with the primers: *tekt2* RT-F: 5′- CAG​CAC​CCG​GAA​ACT​TAG​AGA -3′ and *tekt2* RT-R: 5′-TAT​TGT​ACC​GGC​TGA​ACT​GCT -3′. Quantitative real-time PCR (qRT-PCR) was performed in a total reaction volume of 20 μL containing 10 μL of ChamQ SYBR qPCR Master Mix (Q341-02, Vazyme). The following primers were used to detect *tekt2*: *tekt2* qRT-F: 5′-AAA​TGG​ACG​CTC​TCA​CTC​TG-3′ and *tekt2* qRT-R: 5′-TTC​CCA​CGA​CGG​CCT​TCC​CT-3′. Zebrafish *elongation factor 1a* was used as the internal control, with the following primers: *ef1a* qRT-F: 5′-CTT​CAA​CGC​TCA​GGT​CAT​CA -3′ and *ef1a* qRT-R: 5′-CGG​TCG​ATC​TTC​TCC​TTG​AG-3′. Three independent biological replicates were analyzed, with each sample measured in technical triplicate.

### Plasmid construction

The c5h11orf1 promoter was selected based on our previously published zebrafish single-cell RNA-sequencing dataset, in which c5h11orf1 showed strong enrichment in sensory HCs (Qian et al., 2022). A 2921 bp genomic fragment of the c5h11orf1 promoter was amplified from zebrafish genomic DNA and used to drive expression of the Tekt2-mCherry fusion protein. The c5h11orf1 promoter fragment, tekt2 coding sequence, and mCherry sequence were assembled into the pDestTol2pA2 vector using homologous recombination-based cloning. The resulting construct was injected into one-cell-stage *Tg(pou4f3:mEGFP)* embryos.

### Startle response experiment

The startle-response assay was conducted in a quiet, dark environment. Each experimental group consisted of 50 embryos per dish. At 5 dpf, larvae without visible pericardial edema, body curvature, or other overt morphological abnormalities were randomly selected and transferred to a Petri dish mounted on a mini vibrator. Mechanical stimuli were delivered using the following parameters: 20 cycles, amplitude of 9 dB re. m·s^-2^, frequency of 600 Hz, duration of 30 m, and an inter-stimulus interval of 180 s. Larval movements were recorded with an infrared camera, and the startle response was quantified by calculating the mean moving distance and peak velocity.

### Vestibulo-ocular reflex

The vestibulo-ocular reflex (VOR) assay was performed as previously described. Briefly, 6 dpf larvae without visible pericardial edema, body curvature, or other overt morphological abnormalities were gently immobilized in a larva-shaped chamber containing 6% methylcellulose. The chamber was mounted on the VOR apparatus, and sinusoidal rotations with an amplitude of ±60° were applied. Eye movements were recorded with an infrared camera and analyzed using custom image-analysis software. VOR performance was quantified as the ratio of the maximal change in the projected eye area to the maximal projected eye area in the frontal plane of the larva.

### Dye uptake and phalloidin staining assay

FM4-64 styryl dyes were used to assess HC mechanotransduction activity as previously described. Briefly, 5 dpf zebrafish larvae were exposed to 1 μM FM4-64 (T13320, Invitrogen) for 15 s, followed by three immediate washes in medium containing 0.17 mg/mL tricaine (Sigma) to terminate dye uptake. Images were acquired on a confocal microscope using 561 nm excitation for FM4-64. For stereocilia labeling, iFluor™ 488 phalloidin was used. Larvae were anesthetized and fixed in 4% paraformaldehyde (PFA) either overnight at 4 °C or for 2 h at room temperature. After three washes in 0.1% PBST (PBS with 0.1% Triton X-100; 10 min each), samples were incubated with 200 nM phalloidin for 30 min at 37 °C in the dark, washed three times in 0.1% PBST (10 min each), embedded in low-melting-point agarose, and imaged by confocal microscopy.

### Confocal microscopy

Samples were embedded in 0.85% low-melting-point agarose containing tricaine in glass-bottom dishes. Larval position and orientation were carefully adjusted using a fine needle. Images were acquired on a Nikon confocal microscope (A1R HD25, Nikon) using either a 60×/1.27 NA water-immersion objective or a ×4/0.20 NA air objective. Confocal z-stacks were collected with a 1 μm step size. Imaging parameters were kept identical across experimental groups to ensure comparability. Fluorescence intensity profiles were extracted using Nikon NIS-Elements software.

Live imaging of motile cilia was performed using the Nikon confocal microscope with a ×60 objective. Time-lapse images were acquired continuously using a scan format of 256 × 256 pixels, without an additional delay between consecutive frames. A single focal plane was imaged, and no z-stack was acquired. Each recording consisted of 152 frames acquired over an actual duration of 10 s. The acquisition time and frame number were obtained from the original microscope files. Ciliary beat frequency was calculated by counting the number of complete beating cycles during the actual recording period. The angular excursion of each cilium was defined as the angle between its two extreme positions during beating. Movies were exported at 24 frames per second for presentation. Therefore, the playback duration of the exported movie was approximately 6 s and did not represent the actual acquisition duration used for quantitative analysis.

### Scanning electron microscopy

After anesthesia, 4 dpf larvae were collected and fixed in 2.5% glutaraldehyde at 4 °C overnight. Samples were then washed three times in 0.1 M phosphate buffer (pH 7.0) for 15 min. Specimens were post-fixed in 1% osmium tetroxide for 2 h, followed by three additional washes in phosphate buffer (15 min each). After dehydration through a graded ethanol series, samples were incubated in a 1:1 mixture of ethanol and isoamyl acetate for 30 min and then transferred to 100% isoamyl acetate for 1 h. Specimens were subsequently critical point–dried, sputter-coated, and imaged using a scanning electron microscope (Hitachi SU8100, Japan) at an accelerating voltage of 3.0 kV.

### Statistical analysis

All statistical analyses were performed using GraphPad Prism 8, and data are presented as mean ± standard error of the mean (SEM). For comparisons between two groups, statistical significance was determined using an unpaired two-tailed Student’s t-test. For comparisons among multiple groups, one-way ANOVA followed by Tukey’s multiple-comparisons test or Brown–Forsythe and Welch ANOVA were applied, as applicable. A p-value < 0.05 was considered statistically significant.

## Data Availability

The datasets presented in this study can be found in online repositories. The names of the repository/repositories and accession number(s) can be found in the article/Supplementary Material.

## Supplementary material

SUPPLEMENTARY MATERIAL MOVIE S1 Time-lapse recording of motile cilia activity in the zebrafish otic vesicle. Representative live imaging of the otic vesicle in control and *tekt2* mutant embryos.
